# Supplementary material for: Hot spot prediction in protein-protein interactions by an ensemble system
Source: BMC Syst Biol. 2018 Dec 31;12(Suppl 9):132. doi: 10.1186/s12918-018-0665-8 (PMC6311905; doi:10.1186/s12918-018-0665-8)
Supplement: Supplementary file 2 — All relevant MATLAB code. A simple Matlab implement of our predictor. (ZIP 19,742 kb) [file 12918_2018_665_MOESM2_ESM.zip › Additional file 2/Read ASA/ASA website/BID/1JPPB.html]

 NetSurfP - Protein Surface Accessibility and Secondary Structure Predictions

  

|  |  |  |
| --- | --- | --- |
|  |  | NetSurfP - Protein Surface Accessibility and Secondary Structure Predictions  Technical University of Denmark |

  


---

```
# For publication of results, please cite:
# A generic method for assignment of reliability scores applied to solvent accessibility predictions.
# Bent Petersen, Thomas Nordahl Petersen, Pernille Andersen, Morten Nielsen and Claus Lundegaard
# BMC Structural Biology 2009, 9:51 doi:10.1186/1472-6807-9-51
#
# Column 1: Class assignment - B for buried or E for Exposed - Threshold: 25% exposure, but not based on RSA
# Column 2: Amino acid
# Column 3: Sequence name
# Column 4: Amino acid number
# Column 5: Relative Surface Accessibility - RSA
# Column 6: Absolute Surface Accessibility
# Column 7: Z-fit score for RSA prediction
# Column 8: Probability for Alpha-Helix
# Column 9: Probability for Beta-strand
# Column 10: Probability for Coil
E H  1JPPB                  1    0.627 114.124   0.487   0.018   0.019   0.964
B A  1JPPB                  2    0.193  21.269  -0.446   0.879   0.010   0.111
B V  1JPPB                  3    0.110  16.830  -1.045   0.938   0.007   0.055
B V  1JPPB                  4    0.219  33.645   1.090   0.938   0.007   0.055
E N  1JPPB                  5    0.368  53.861   0.590   0.938   0.007   0.055
B L  1JPPB                  6    0.077  14.117   0.986   0.879   0.010   0.111
B I  1JPPB                  7    0.099  18.315  -0.023   0.717   0.014   0.269
E N  1JPPB                  8    0.502  73.566   0.158   0.600   0.003   0.397
E Y  1JPPB                  9    0.389  83.108   0.338   0.600   0.003   0.397
E Q  1JPPB                 10    0.379  67.672  -0.381   0.502   0.002   0.495
E D  1JPPB                 11    0.498  71.690   0.050   0.600   0.003   0.397
E D  1JPPB                 12    0.549  79.140   0.699   0.858   0.002   0.139
E A  1JPPB                 13    0.431  47.518   0.964   0.923   0.002   0.076
E E  1JPPB                 14    0.295  51.467   0.787   0.923   0.002   0.076
B L  1JPPB                 15    0.155  28.399   0.938   0.879   0.010   0.111
E A  1JPPB                 16    0.515  56.709   0.370   0.858   0.002   0.139
E T  1JPPB                 17    0.447  61.957   0.464   0.694   0.003   0.303
E R  1JPPB                 18    0.459 105.180   0.260   0.406   0.004   0.590
B A  1JPPB                 19    0.071   7.824   0.203   0.694   0.003   0.303
B I  1JPPB                 20    0.046   8.492   0.693   0.923   0.002   0.076
E P  1JPPB                 21    0.489  69.375   1.870   0.970   0.001   0.030
E E  1JPPB                 22    0.318  55.642   1.645   0.970   0.001   0.030
B L  1JPPB                 23    0.021   3.863   1.266   0.988   0.000   0.012
B T  1JPPB                 24    0.118  16.339   1.093   0.970   0.001   0.030
E K  1JPPB                 25    0.558 114.781   1.617   0.970   0.001   0.030
B L  1JPPB                 26    0.141  25.799   0.557   0.858   0.002   0.139
B L  1JPPB                 27    0.045   8.185  -0.221   0.502   0.002   0.495
E N  1JPPB                 28    0.626  91.676   0.740   0.176   0.004   0.820
E D  1JPPB                 29    0.384  55.277   0.113   0.016   0.005   0.979
E E  1JPPB                 30    0.779 136.021  -0.917   0.003   0.003   0.994
E D  1JPPB                 31    0.478  68.937   0.227   0.003   0.003   0.994
E Q  1JPPB                 32    0.563 100.605   0.860   0.970   0.001   0.030
E V  1JPPB                 33    0.602  92.543   1.390   0.975   0.003   0.022
B V  1JPPB                 34    0.079  12.127   0.995   0.975   0.003   0.022
B V  1JPPB                 35    0.076  11.727   1.279   0.970   0.001   0.030
E N  1JPPB                 36    0.342  50.025   1.729   0.970   0.001   0.030
E K  1JPPB                 37    0.331  68.169   1.960   0.970   0.001   0.030
B A  1JPPB                 38    0.016   1.763   1.413   0.970   0.001   0.030
B A  1JPPB                 39    0.026   2.920   0.486   0.970   0.001   0.030
E V  1JPPB                 40    0.366  56.316   1.572   0.988   0.000   0.012
B M  1JPPB                 41    0.074  14.807   0.913   0.988   0.000   0.012
B V  1JPPB                 42    0.017   2.644   1.400   0.988   0.000   0.012
B H  1JPPB                 43    0.167  30.323   0.286   0.970   0.001   0.030
E Q  1JPPB                 44    0.384  68.582   1.404   0.970   0.001   0.030
B L  1JPPB                 45    0.048   8.862   0.648   0.938   0.007   0.055
B S  1JPPB                 46    0.040   4.747  -2.137   0.717   0.014   0.269
E K  1JPPB                 47    0.541 111.222   0.689   0.246   0.004   0.750
E K  1JPPB                 48    0.499 102.706  -0.212   0.053   0.005   0.942
E E  1JPPB                 49    0.538  93.936  -0.249   0.053   0.005   0.942
E A  1JPPB                 50    0.764  84.160  -0.596   0.600   0.003   0.397
E S  1JPPB                 51    0.574  67.214   0.615   0.858   0.002   0.139
B R  1JPPB                 52    0.148  33.846   0.502   0.938   0.007   0.055
E H  1JPPB                 53    0.346  63.010   1.237   0.938   0.007   0.055
E A  1JPPB                 54    0.490  54.009   1.360   0.975   0.003   0.022
B I  1JPPB                 55    0.158  29.249   0.554   0.938   0.007   0.055
B M  1JPPB                 56    0.079  15.728  -0.962   0.938   0.007   0.055
E R  1JPPB                 57    0.495 113.332   0.025   0.879   0.010   0.111
E S  1JPPB                 58    0.568  66.616  -0.568   0.600   0.003   0.397
E P  1JPPB                 59    0.395  56.036  -0.443   0.321   0.003   0.675
E Q  1JPPB                 60    0.554  98.927  -0.544   0.109   0.005   0.886
B M  1JPPB                 61    0.075  15.048   0.062   0.600   0.003   0.397
B V  1JPPB                 62    0.050   7.608   0.695   0.938   0.007   0.055
E S  1JPPB                 63    0.444  52.060   1.464   0.975   0.003   0.022
B A  1JPPB                 64    0.263  28.961   1.426   0.975   0.003   0.022
B I  1JPPB                 65    0.022   3.977   1.063   0.988   0.000   0.012
B V  1JPPB                 66    0.091  13.956   0.698   0.988   0.000   0.012
E R  1JPPB                 67    0.531 121.599   1.857   0.970   0.001   0.030
B T  1JPPB                 68    0.133  18.461   0.742   0.923   0.002   0.076
B M  1JPPB                 69    0.026   5.283   0.020   0.694   0.003   0.303
E Q  1JPPB                 70    0.481  85.889   0.650   0.321   0.003   0.675
E N  1JPPB                 71    0.649  94.955   0.898   0.176   0.004   0.820
E T  1JPPB                 72    0.579  80.349  -1.087   0.016   0.005   0.979
E N  1JPPB                 73    0.722 105.730  -1.253   0.003   0.003   0.994
E D  1JPPB                 74    0.437  62.900   0.527   0.003   0.003   0.994
E V  1JPPB                 75    0.516  79.355   0.469   0.923   0.002   0.076
E E  1JPPB                 76    0.551  96.207   1.500   0.970   0.001   0.030
B T  1JPPB                 77    0.068   9.404   0.388   0.975   0.003   0.022
B A  1JPPB                 78    0.074   8.144   1.116   0.970   0.001   0.030
E R  1JPPB                 79    0.312  71.334   1.107   0.970   0.001   0.030
E C  1JPPB                 80    0.322  45.237   1.579   0.970   0.001   0.030
B T  1JPPB                 81    0.017   2.400   1.466   0.970   0.001   0.030
B A  1JPPB                 82    0.028   3.119   0.142   0.970   0.001   0.030
E G  1JPPB                 83    0.319  25.082   1.381   0.970   0.001   0.030
B T  1JPPB                 84    0.060   8.280   0.741   0.988   0.000   0.012
B L  1JPPB                 85    0.017   3.186   1.403   0.988   0.000   0.012
B H  1JPPB                 86    0.211  38.290   0.840   0.970   0.001   0.030
E N  1JPPB                 87    0.438  64.167   1.296   0.923   0.002   0.076
B L  1JPPB                 88    0.066  12.030   0.472   0.782   0.003   0.216
B S  1JPPB                 89    0.069   8.075  -2.289   0.321   0.003   0.675
E H  1JPPB                 90    0.566 102.919  -0.094   0.053   0.005   0.942
E H  1JPPB                 91    0.428  77.817   0.109   0.016   0.005   0.979
E R  1JPPB                 92    0.649 148.621  -0.127   0.600   0.003   0.397
E E  1JPPB                 93    0.771 134.624   0.762   0.782   0.003   0.216
B G  1JPPB                 94    0.116   9.145   0.019   0.879   0.010   0.111
B L  1JPPB                 95    0.123  22.430   0.849   0.923   0.002   0.076
E L  1JPPB                 96    0.502  91.916   1.718   0.970   0.001   0.030
E A  1JPPB                 97    0.261  28.817   1.805   0.970   0.001   0.030
B I  1JPPB                 98    0.026   4.828   0.350   0.970   0.001   0.030
B F  1JPPB                 99    0.205  41.184   0.647   0.938   0.007   0.055
E K  1JPPB                100    0.715 146.993   0.856   0.802   0.014   0.185
E S  1JPPB                101    0.279  32.734   0.502   0.321   0.003   0.675
E G  1JPPB                102    0.492  38.728  -1.149   0.109   0.005   0.886
B G  1JPPB                103    0.081   6.406   0.198   0.246   0.004   0.750
B I  1JPPB                104    0.059  11.007   0.913   0.858   0.002   0.139
E P  1JPPB                105    0.436  61.911   1.211   0.938   0.007   0.055
B A  1JPPB                106    0.238  26.272   1.359   0.975   0.003   0.022
B L  1JPPB                107    0.017   3.076   1.340   0.970   0.001   0.030
B V  1JPPB                108    0.108  16.523   0.730   0.970   0.001   0.030
E K  1JPPB                109    0.507 104.187   1.608   0.970   0.001   0.030
B M  1JPPB                110    0.091  18.269   0.639   0.858   0.002   0.139
B L  1JPPB                111    0.043   7.837  -0.226   0.600   0.003   0.397
E G  1JPPB                112    0.639  50.297   1.075   0.176   0.004   0.820
E S  1JPPB                113    0.476  55.822   0.203   0.016   0.005   0.979
E P  1JPPB                114    0.712 101.004  -0.624   0.003   0.003   0.994
E V  1JPPB                115    0.492  75.590   0.259   0.003   0.003   0.994
E D  1JPPB                116    0.600  86.460   0.920   0.923   0.002   0.076
E S  1JPPB                117    0.561  65.702   1.667   0.970   0.001   0.030
B V  1JPPB                118    0.052   8.054   0.607   0.975   0.003   0.022
B L  1JPPB                119    0.085  15.618   1.157   0.970   0.001   0.030
E F  1JPPB                120    0.330  66.191   0.998   0.970   0.001   0.030
E Y  1JPPB                121    0.261  55.776   1.497   0.970   0.001   0.030
B A  1JPPB                122    0.017   1.873   1.281   0.970   0.001   0.030
B I  1JPPB                123    0.031   5.717  -0.157   0.970   0.001   0.030
E T  1JPPB                124    0.272  37.782   0.572   0.970   0.001   0.030
B T  1JPPB                125    0.048   6.602   0.895   0.988   0.000   0.012
B L  1JPPB                126    0.017   3.149   1.235   0.988   0.000   0.012
B H  1JPPB                127    0.145  26.357   0.233   0.970   0.001   0.030
E N  1JPPB                128    0.348  50.903   0.262   0.923   0.002   0.076
B L  1JPPB                129    0.077  14.172   0.565   0.858   0.002   0.139
B L  1JPPB                130    0.044   8.075  -1.469   0.502   0.002   0.495
E L  1JPPB                131    0.455  83.329  -0.350   0.109   0.005   0.886
E H  1JPPB                132    0.532  96.680  -0.420   0.016   0.005   0.979
E Q  1JPPB                133    0.489  87.282  -0.034   0.109   0.005   0.886
E E  1JPPB                134    0.807 141.000  -0.325   0.858   0.002   0.139
E G  1JPPB                135    0.581  45.756   0.652   0.858   0.002   0.139
B A  1JPPB                136    0.128  14.150   0.012   0.923   0.002   0.076
E K  1JPPB                137    0.372  76.479   1.197   0.970   0.001   0.030
E M  1JPPB                138    0.494  98.869   1.210   0.970   0.001   0.030
B A  1JPPB                139    0.204  22.459   1.111   0.970   0.001   0.030
B V  1JPPB                140    0.061   9.345  -0.395   0.923   0.002   0.076
E R  1JPPB                141    0.356  81.410   0.512   0.879   0.010   0.111
E L  1JPPB                142    0.626 114.639   0.002   0.782   0.003   0.216
E A  1JPPB                143    0.412  45.402   0.240   0.321   0.003   0.675
E G  1JPPB                144    0.560  44.080  -0.802   0.176   0.004   0.820
B G  1JPPB                145    0.086   6.776  -0.151   0.321   0.003   0.675
B L  1JPPB                146    0.067  12.176   0.360   0.782   0.003   0.216
E Q  1JPPB                147    0.465  82.995   1.135   0.938   0.007   0.055
E K  1JPPB                148    0.271  55.786   1.082   0.975   0.003   0.022
B M  1JPPB                149    0.025   5.083   0.700   0.970   0.001   0.030
B V  1JPPB                150    0.097  14.894   0.485   0.970   0.001   0.030
E A  1JPPB                151    0.514  56.610   1.466   0.975   0.003   0.022
B L  1JPPB                152    0.102  18.695   0.156   0.858   0.002   0.139
B L  1JPPB                153    0.062  11.425  -0.575   0.694   0.003   0.303
E N  1JPPB                154    0.629  92.130   0.464   0.321   0.003   0.675
E K  1JPPB                155    0.520 107.005  -0.068   0.053   0.005   0.942
E T  1JPPB                156    0.709  98.311  -2.080   0.003   0.003   0.994
E N  1JPPB                157    0.421  61.664  -0.593   0.003   0.003   0.994
E V  1JPPB                158    0.525  80.754  -0.106   0.858   0.002   0.139
E K  1JPPB                159    0.521 107.087   0.949   0.923   0.002   0.076
B F  1JPPB                160    0.073  14.691  -0.398   0.923   0.002   0.076
B L  1JPPB                161    0.146  26.678   1.132   0.970   0.001   0.030
E A  1JPPB                162    0.404  44.554   0.611   0.970   0.001   0.030
B I  1JPPB                163    0.229  42.347   0.649   0.970   0.001   0.030
B T  1JPPB                164    0.035   4.841  -0.151   0.970   0.001   0.030
B T  1JPPB                165    0.082  11.373  -0.443   0.970   0.001   0.030
E D  1JPPB                166    0.348  50.147   0.600   0.970   0.001   0.030
B C  1JPPB                167    0.057   7.989   0.327   0.970   0.001   0.030
B L  1JPPB                168    0.034   6.280  -0.529   0.970   0.001   0.030
B Q  1JPPB                169    0.240  42.846   0.408   0.923   0.002   0.076
E I  1JPPB                170    0.396  73.205  -0.075   0.858   0.002   0.139
B L  1JPPB                171    0.126  23.126   0.234   0.782   0.003   0.216
B A  1JPPB                172    0.117  12.893  -2.747   0.406   0.004   0.590
E Y  1JPPB                173    0.463  98.858  -0.585   0.176   0.004   0.820
E G  1JPPB                174    0.578  45.473  -1.164   0.053   0.005   0.942
E N  1JPPB                175    0.542  79.363  -0.730   0.109   0.005   0.886
E Q  1JPPB                176    0.676 120.716  -1.000   0.782   0.003   0.216
E E  1JPPB                177    0.596 104.156   0.266   0.782   0.003   0.216
B S  1JPPB                178    0.140  16.361  -0.103   0.782   0.003   0.216
E K  1JPPB                179    0.282  58.007   0.834   0.923   0.002   0.076
E L  1JPPB                180    0.514  94.022   0.529   0.923   0.002   0.076
B I  1JPPB                181    0.224  41.385   0.538   0.923   0.002   0.076
B I  1JPPB                182    0.083  15.447  -1.304   0.938   0.007   0.055
E L  1JPPB                183    0.375  68.699  -0.151   0.879   0.010   0.111
E A  1JPPB                184    0.612  67.420  -0.282   0.717   0.014   0.269
E S  1JPPB                185    0.398  46.610  -0.090   0.406   0.004   0.590
E G  1JPPB                186    0.560  44.048  -0.719   0.176   0.004   0.820
B G  1JPPB                187    0.091   7.154  -0.166   0.406   0.004   0.590
B P  1JPPB                188    0.068   9.678   0.148   0.858   0.002   0.139
E Q  1JPPB                189    0.450  80.370   1.134   0.938   0.007   0.055
B A  1JPPB                190    0.236  25.963   0.723   0.975   0.003   0.022
B L  1JPPB                191    0.027   4.944   0.309   0.970   0.001   0.030
B V  1JPPB                192    0.086  13.280   0.527   0.970   0.001   0.030
E N  1JPPB                193    0.482  70.565   1.574   0.970   0.001   0.030
B I  1JPPB                194    0.093  17.131   0.304   0.858   0.002   0.139
B M  1JPPB                195    0.045   9.105  -0.186   0.694   0.003   0.303
E R  1JPPB                196    0.531 121.668   0.492   0.406   0.004   0.590
E T  1JPPB                197    0.578  80.224   0.135   0.176   0.004   0.820
E Y  1JPPB                198    0.586 125.250  -1.465   0.053   0.005   0.942
E T  1JPPB                199    0.666  92.333  -0.992   0.003   0.003   0.994
E Y  1JPPB                200    0.375  80.202   0.160   0.246   0.004   0.750
E E  1JPPB                201    0.672 117.416   0.366   0.923   0.002   0.076
E K  1JPPB                202    0.506 104.105   1.295   0.923   0.002   0.076
B L  1JPPB                203    0.065  11.810   0.145   0.970   0.001   0.030
B L  1JPPB                204    0.137  25.085   1.053   0.970   0.001   0.030
E W  1JPPB                205    0.405  97.330   1.021   0.970   0.001   0.030
B T  1JPPB                206    0.157  21.776   0.941   0.970   0.001   0.030
B T  1JPPB                207    0.015   2.025   1.483   0.970   0.001   0.030
B S  1JPPB                208    0.083   9.774   0.027   0.970   0.001   0.030
E R  1JPPB                209    0.268  61.326   0.862   0.970   0.001   0.030
B V  1JPPB                210    0.026   3.981   0.860   0.988   0.000   0.012
B L  1JPPB                211    0.029   5.255   0.424   0.970   0.001   0.030
B K  1JPPB                212    0.260  53.400   0.316   0.970   0.001   0.030
B V  1JPPB                213    0.259  39.793   0.332   0.923   0.002   0.076
B L  1JPPB                214    0.046   8.404   0.005   0.782   0.003   0.216
B S  1JPPB                215    0.153  17.955  -1.969   0.406   0.004   0.590
E V  1JPPB                216    0.537  82.598  -0.691   0.109   0.005   0.886
E C  1JPPB                217    0.361  50.642  -0.066   0.018   0.019   0.964
E S  1JPPB                218    0.659  77.247  -0.712   0.257   0.016   0.727
E S  1JPPB                219    0.758  88.861  -0.386   0.502   0.002   0.495
E N  1JPPB                220    0.292  42.720  -0.556   0.600   0.003   0.397
E K  1JPPB                221    0.283  58.254   0.613   0.782   0.003   0.216
E P  1JPPB                222    0.585  83.040   0.307   0.802   0.014   0.185
E A  1JPPB                223    0.423  46.582   0.335   0.802   0.014   0.185
E I  1JPPB                224    0.350  64.824  -0.856   0.802   0.014   0.185
B V  1JPPB                225    0.257  39.486  -0.529   0.802   0.014   0.185
E E  1JPPB                226    0.549  95.893  -0.555   0.717   0.014   0.269
E A  1JPPB                227    0.421  46.438  -0.457   0.622   0.015   0.363
E G  1JPPB                228    0.536  42.175  -0.785   0.502   0.002   0.495
B G  1JPPB                229    0.067   5.241  -0.725   0.694   0.003   0.303
B M  1JPPB                230    0.112  22.511   0.558   0.923   0.002   0.076
E Q  1JPPB                231    0.506  90.336   0.503   0.970   0.001   0.030
B A  1JPPB                232    0.250  27.583   0.590   0.970   0.001   0.030
B L  1JPPB                233    0.040   7.269  -0.474   0.970   0.001   0.030
B G  1JPPB                234    0.199  15.669   0.288   0.970   0.001   0.030
E L  1JPPB                235    0.543  99.405   0.923   0.923   0.002   0.076
B H  1JPPB                236    0.170  30.850  -0.333   0.782   0.003   0.216
B L  1JPPB                237    0.110  20.086  -0.990   0.502   0.002   0.495
E T  1JPPB                238    0.675  93.595   0.083   0.246   0.004   0.750
E D  1JPPB                239    0.479  68.995  -0.489   0.016   0.005   0.979
E P  1JPPB                240    0.664  94.222  -1.399   0.016   0.005   0.979
E S  1JPPB                241    0.594  69.617  -0.305   0.016   0.005   0.979
E Q  1JPPB                242    0.601 107.285   0.677   0.923   0.002   0.076
E R  1JPPB                243    0.578 132.293   1.198   0.923   0.002   0.076
B L  1JPPB                244    0.080  14.685   0.452   0.970   0.001   0.030
B V  1JPPB                245    0.108  16.646   0.837   0.970   0.001   0.030
E Q  1JPPB                246    0.388  69.243   1.057   0.970   0.001   0.030
E N  1JPPB                247    0.295  43.159   1.177   0.970   0.001   0.030
B C  1JPPB                248    0.020   2.836   0.880   0.970   0.001   0.030
B L  1JPPB                249    0.053   9.686   0.120   0.970   0.001   0.030
E W  1JPPB                250    0.311  74.747   0.898   0.970   0.001   0.030
B T  1JPPB                251    0.048   6.602   0.032   0.970   0.001   0.030
B L  1JPPB                252    0.036   6.628  -0.647   0.970   0.001   0.030
B R  1JPPB                253    0.229  52.372  -0.026   0.923   0.002   0.076
E N  1JPPB                254    0.373  54.578   0.156   0.858   0.002   0.139
B L  1JPPB                255    0.068  12.432  -0.143   0.600   0.003   0.397
B S  1JPPB                256    0.228  26.722  -1.497   0.321   0.003   0.675
E D  1JPPB                257    0.604  87.080  -0.635   0.246   0.004   0.750
E A  1JPPB                258    0.433  47.728  -0.996   0.176   0.004   0.820
E A  1JPPB                259    0.543  59.795  -1.884   0.321   0.003   0.675
E T  1JPPB                260    0.615  85.273  -0.902   0.600   0.003   0.397
E K  1JPPB                261    0.410  84.358  -0.287   0.782   0.003   0.216
E Q  1JPPB                262    0.328  58.617  -0.069   0.782   0.003   0.216
E E  1JPPB                263    0.407  71.103  -0.547   0.858   0.002   0.139
E G  1JPPB                264    0.334  26.325  -0.832   0.858   0.002   0.139
B M  1JPPB                265    0.192  38.339  -0.688   0.858   0.002   0.139
E E  1JPPB                266    0.370  64.604  -0.385   0.858   0.002   0.139
E G  1JPPB                267    0.494  38.838  -0.351   0.694   0.003   0.303
B L  1JPPB                268    0.115  21.057  -0.642   0.782   0.003   0.216
B L  1JPPB                269    0.079  14.410   0.227   0.923   0.002   0.076
E G  1JPPB                270    0.453  35.643   0.409   0.970   0.001   0.030
B T  1JPPB                271    0.231  32.012   0.837   0.970   0.001   0.030
B L  1JPPB                272    0.033   6.134  -0.178   0.970   0.001   0.030
B V  1JPPB                273    0.161  24.807   0.421   0.970   0.001   0.030
E Q  1JPPB                274    0.506  90.372   1.066   0.923   0.002   0.076
B L  1JPPB                275    0.120  22.027  -0.544   0.782   0.003   0.216
B L  1JPPB                276    0.106  19.335  -0.697   0.502   0.002   0.495
E G  1JPPB                277    0.657  51.714   0.235   0.321   0.003   0.675
E S  1JPPB                278    0.516  60.463  -0.485   0.109   0.005   0.886
E D  1JPPB                279    0.644  92.800  -2.100   0.016   0.005   0.979
E D  1JPPB                280    0.476  68.534  -0.351   0.016   0.005   0.979
E I  1JPPB                281    0.580 107.318   0.453   0.858   0.002   0.139
E N  1JPPB                282    0.579  84.707   1.109   0.923   0.002   0.076
B V  1JPPB                283    0.077  11.866   0.006   0.938   0.007   0.055
B V  1JPPB                284    0.144  22.164   1.063   0.970   0.001   0.030
E T  1JPPB                285    0.451  62.554   0.967   0.970   0.001   0.030
E C  1JPPB                286    0.273  38.357   1.178   0.970   0.001   0.030
B A  1JPPB                287    0.020   2.226   0.757   0.970   0.001   0.030
B A  1JPPB                288    0.105  11.582  -0.536   0.970   0.001   0.030
E G  1JPPB                289    0.387  30.465   0.697   0.970   0.001   0.030
B I  1JPPB                290    0.041   7.585  -0.344   0.970   0.001   0.030
B L  1JPPB                291    0.039   7.214  -0.954   0.970   0.001   0.030
E S  1JPPB                292    0.270  31.679   0.096   0.923   0.002   0.076
E N  1JPPB                293    0.365  53.465  -0.215   0.858   0.002   0.139
B L  1JPPB                294    0.071  13.037  -0.219   0.694   0.003   0.303
E T  1JPPB                295    0.313  43.413  -0.880   0.502   0.002   0.495
E C  1JPPB                296    0.534  74.960  -0.990   0.246   0.004   0.750
E N  1JPPB                297    0.548  80.213  -1.144   0.109   0.005   0.886
E N  1JPPB                298    0.409  59.863  -0.463   0.109   0.005   0.886
E Y  1JPPB                299    0.671 143.500  -0.095   0.858   0.002   0.139
E K  1JPPB                300    0.678 139.567   0.823   0.858   0.002   0.139
B N  1JPPB                301    0.158  23.117  -0.019   0.923   0.002   0.076
B K  1JPPB                302    0.221  45.398   0.673   0.970   0.001   0.030
E M  1JPPB                303    0.536 107.194   1.007   0.923   0.002   0.076
E M  1JPPB                304    0.298  59.550   1.202   0.923   0.002   0.076
B V  1JPPB                305    0.064   9.898  -1.141   0.858   0.002   0.139
E C  1JPPB                306    0.304  42.611  -0.406   0.858   0.002   0.139
E Q  1JPPB                307    0.579 103.392  -0.186   0.782   0.003   0.216
E V  1JPPB                308    0.463  71.240  -0.649   0.694   0.003   0.303
E G  1JPPB                309    0.410  32.298  -0.543   0.600   0.003   0.397
B G  1JPPB                310    0.263  20.667  -0.321   0.406   0.004   0.590
B I  1JPPB                311    0.096  17.741   0.443   0.782   0.003   0.216
E E  1JPPB                312    0.485  84.677   0.730   0.923   0.002   0.076
E A  1JPPB                313    0.308  33.975   0.616   0.970   0.001   0.030
B L  1JPPB                314    0.038   6.994  -0.031   0.970   0.001   0.030
B V  1JPPB                315    0.092  14.156   0.427   0.970   0.001   0.030
E R  1JPPB                316    0.487 111.523   1.357   0.970   0.001   0.030
B T  1JPPB                317    0.116  16.048   0.147   0.923   0.002   0.076
B V  1JPPB                318    0.044   6.747  -0.253   0.858   0.002   0.139
E L  1JPPB                319    0.469  85.947   0.380   0.600   0.003   0.397
E R  1JPPB                320    0.668 152.995   0.530   0.321   0.003   0.675
E A  1JPPB                321    0.523  57.602  -1.457   0.053   0.005   0.942
E G  1JPPB                322    0.579  45.567  -1.660   0.003   0.003   0.994
E D  1JPPB                323    0.551  79.428  -0.828   0.016   0.005   0.979
E R  1JPPB                324    0.487 111.592   0.625   0.600   0.003   0.397
E E  1JPPB                325    0.569  99.422  -0.247   0.923   0.002   0.076
E D  1JPPB                326    0.496  71.488   1.288   0.970   0.001   0.030
B I  1JPPB                327    0.098  18.204   0.554   0.970   0.001   0.030
B T  1JPPB                328    0.203  28.128   1.108   0.970   0.001   0.030
E E  1JPPB                329    0.437  76.396   0.732   0.970   0.001   0.030
B P  1JPPB                330    0.235  33.290   0.919   0.970   0.001   0.030
B A  1JPPB                331    0.032   3.559  -0.311   0.970   0.001   0.030
B I  1JPPB                332    0.085  15.669  -0.447   0.923   0.002   0.076
B C  1JPPB                333    0.190  26.662   0.233   0.923   0.002   0.076
B A  1JPPB                334    0.030   3.306  -0.299   0.923   0.002   0.076
B L  1JPPB                335    0.148  27.044   0.059   0.923   0.002   0.076
B R  1JPPB                336    0.255  58.281  -0.644   0.858   0.002   0.139
B H  1JPPB                337    0.201  36.616  -0.847   0.782   0.003   0.216
B L  1JPPB                338    0.138  25.268  -0.641   0.694   0.003   0.303
B T  1JPPB                339    0.226  31.388  -1.947   0.502   0.002   0.495
E S  1JPPB                340    0.357  41.805  -1.373   0.321   0.003   0.675
E R  1JPPB                341    0.426  97.668  -1.026   0.246   0.004   0.750
E H  1JPPB                342    0.437  79.454  -1.423   0.109   0.005   0.886
E Q  1JPPB                343    0.616 109.946  -1.916   0.246   0.004   0.750
E E  1JPPB                344    0.519  90.669  -1.295   0.246   0.004   0.750
E A  1JPPB                345    0.355  39.110  -0.135   0.600   0.003   0.397
E E  1JPPB                346    0.438  76.571  -0.209   0.782   0.003   0.216
E M  1JPPB                347    0.430  86.063   0.461   0.782   0.003   0.216
B A  1JPPB                348    0.167  18.370  -0.692   0.858   0.002   0.139
E Q  1JPPB                349    0.281  50.258   0.242   0.858   0.002   0.139
E N  1JPPB                350    0.507  74.254   0.080   0.858   0.002   0.139
B A  1JPPB                351    0.205  22.591   0.095   0.858   0.002   0.139
B V  1JPPB                352    0.165  25.299  -1.359   0.782   0.003   0.216
E R  1JPPB                353    0.443 101.401  -0.373   0.802   0.014   0.185
E L  1JPPB                354    0.552 101.016  -0.386   0.622   0.015   0.363
E H  1JPPB                355    0.444  80.709  -1.259   0.257   0.016   0.727
E Y  1JPPB                356    0.575 122.920  -1.408   0.109   0.005   0.886
B G  1JPPB                357    0.267  21.029  -0.497   0.181   0.016   0.803
B L  1JPPB                358    0.113  20.727   0.276   0.561   0.047   0.393
E P  1JPPB                359    0.461  65.416   0.654   0.831   0.044   0.125
E V  1JPPB                360    0.317  48.738   0.177   0.911   0.033   0.057
B V  1JPPB                361    0.127  19.505   0.116   0.831   0.044   0.125
B V  1JPPB                362    0.146  22.456   0.129   0.831   0.044   0.125
E K  1JPPB                363    0.496 101.986   0.535   0.831   0.044   0.125
B L  1JPPB                364    0.203  37.188   0.161   0.717   0.014   0.269
B L  1JPPB                365    0.087  15.911  -1.030   0.406   0.004   0.590
E H  1JPPB                366    0.559 101.737  -0.057   0.257   0.016   0.727
E P  1JPPB                367    0.533  75.590  -0.164   0.181   0.016   0.803
E P  1JPPB                368    0.595  84.388  -1.764   0.053   0.005   0.942
E S  1JPPB                369    0.672  78.735  -1.104   0.016   0.005   0.979
E H  1JPPB                370    0.371  67.485   0.114   0.502   0.002   0.495
E W  1JPPB                371    0.559 134.536   0.241   0.858   0.002   0.139
E P  1JPPB                372    0.509  72.185   0.428   0.858   0.002   0.139
B L  1JPPB                373    0.168  30.706   0.265   0.923   0.002   0.076
B I  1JPPB                374    0.246  45.547   0.796   0.970   0.001   0.030
E K  1JPPB                375    0.488 100.361   0.808   0.970   0.001   0.030
B A  1JPPB                376    0.240  26.426  -0.151   0.923   0.002   0.076
B T  1JPPB                377    0.134  18.558  -0.055   0.970   0.001   0.030
B V  1JPPB                378    0.173  26.621  -0.390   0.970   0.001   0.030
E G  1JPPB                379    0.271  21.343   0.253   0.970   0.001   0.030
B L  1JPPB                380    0.040   7.287  -0.232   0.970   0.001   0.030
B I  1JPPB                381    0.052   9.583   0.332   0.970   0.001   0.030
B R  1JPPB                382    0.233  53.265  -0.274   0.923   0.002   0.076
B N  1JPPB                383    0.257  37.625  -0.264   0.879   0.010   0.111
B L  1JPPB                384    0.081  14.794  -0.621   0.694   0.003   0.303
B A  1JPPB                385    0.176  19.384  -2.514   0.321   0.003   0.675
E L  1JPPB                386    0.499  91.458  -0.823   0.109   0.005   0.886
E C  1JPPB                387    0.333  46.767  -0.006   0.016   0.005   0.979
E P  1JPPB                388    0.590  83.707  -0.765   0.600   0.003   0.397
E A  1JPPB                389    0.761  83.906   0.523   0.694   0.003   0.303
B N  1JPPB                390    0.198  29.002  -0.162   0.782   0.003   0.216
B H  1JPPB                391    0.181  32.960   0.594   0.858   0.002   0.139
E A  1JPPB                392    0.509  56.136   0.458   0.923   0.002   0.076
E P  1JPPB                393    0.339  48.090   1.177   0.970   0.001   0.030
B L  1JPPB                394    0.073  13.385  -0.939   0.970   0.001   0.030
B R  1JPPB                395    0.244  55.784   0.093   0.938   0.007   0.055
E E  1JPPB                396    0.427  74.562  -0.441   0.879   0.010   0.111
E Q  1JPPB                397    0.371  66.261  -0.121   0.782   0.003   0.216
E G  1JPPB                398    0.416  32.739  -1.219   0.502   0.002   0.495
B A  1JPPB                399    0.110  12.133  -0.458   0.600   0.003   0.397
B I  1JPPB                400    0.088  16.372   0.043   0.923   0.002   0.076
E P  1JPPB                401    0.503  71.347   0.811   0.970   0.001   0.030
E R  1JPPB                402    0.256  58.624   1.044   0.970   0.001   0.030
B L  1JPPB                403    0.024   4.449   0.831   0.970   0.001   0.030
B V  1JPPB                404    0.132  20.242   0.623   0.970   0.001   0.030
E Q  1JPPB                405    0.532  94.962   1.072   0.923   0.002   0.076
B L  1JPPB                406    0.113  20.745  -0.045   0.858   0.002   0.139
B L  1JPPB                407    0.049   8.954  -0.225   0.600   0.003   0.397
E V  1JPPB                408    0.559  85.872   0.327   0.321   0.003   0.675
E R  1JPPB                409    0.491 112.393  -0.160   0.176   0.004   0.820
E A  1JPPB                410    0.598  65.867  -1.494   0.109   0.005   0.886
E H  1JPPB                411    0.591 107.448  -1.694   0.053   0.005   0.942
E Q  1JPPB                412    0.570 101.731  -0.574   0.502   0.002   0.495
E D  1JPPB                413    0.641  92.411  -0.327   0.694   0.003   0.303
E T  1JPPB                414    0.338  46.881   0.136   0.858   0.002   0.139
E Q  1JPPB                415    0.326  58.224  -0.249   0.858   0.002   0.139
E R  1JPPB                416    0.435  99.523  -0.166   0.858   0.002   0.139
E R  1JPPB                417    0.471 107.882   0.043   0.858   0.002   0.139
B T  1JPPB                418    0.295  40.875  -0.705   0.858   0.002   0.139
B S  1JPPB                419    0.266  31.175  -0.775   0.858   0.002   0.139
E M  1JPPB                420    0.369  73.897  -0.555   0.858   0.002   0.139
E G  1JPPB                421    0.348  27.380  -1.104   0.858   0.002   0.139
B G  1JPPB                422    0.262  20.627  -1.222   0.858   0.002   0.139
B T  1JPPB                423    0.193  26.797  -1.113   0.782   0.003   0.216
B Q  1JPPB                424    0.305  54.527  -0.966   0.694   0.003   0.303
E Q  1JPPB                425    0.348  62.117  -1.130   0.694   0.003   0.303
B Q  1JPPB                426    0.288  51.365  -1.290   0.600   0.003   0.397
B F  1JPPB                427    0.228  45.740  -1.601   0.502   0.002   0.495
E V  1JPPB                428    0.426  65.445  -0.905   0.522   0.016   0.462
E E  1JPPB                429    0.506  88.381  -1.345   0.430   0.016   0.555
E G  1JPPB                430    0.443  34.856  -1.490   0.339   0.016   0.645
E V  1JPPB                431    0.379  58.314  -1.471   0.257   0.016   0.727
E R  1JPPB                432    0.567 129.935  -1.279   0.321   0.003   0.675
E M  1JPPB                433    0.371  74.277  -0.609   0.406   0.004   0.590
E E  1JPPB                434    0.503  87.822  -0.780   0.694   0.003   0.303
E E  1JPPB                435    0.553  96.557  -0.865   0.782   0.003   0.216
B I  1JPPB                436    0.193  35.686   0.114   0.782   0.003   0.216
B V  1JPPB                437    0.179  27.528  -0.637   0.923   0.002   0.076
E E  1JPPB                438    0.405  70.719  -0.134   0.923   0.002   0.076
E G  1JPPB                439    0.318  25.034  -0.291   0.858   0.002   0.139
B C  1JPPB                440    0.060   8.396  -0.540   0.923   0.002   0.076
B T  1JPPB                441    0.132  18.350  -0.423   0.970   0.001   0.030
E G  1JPPB                442    0.397  31.252   0.468   0.970   0.001   0.030
B A  1JPPB                443    0.086   9.499   0.133   0.970   0.001   0.030
B L  1JPPB                444    0.053   9.723   0.103   0.923   0.002   0.076
E H  1JPPB                445    0.348  63.265   0.215   0.923   0.002   0.076
E I  1JPPB                446    0.369  68.321   0.204   0.782   0.003   0.216
B L  1JPPB                447    0.118  21.551  -0.032   0.600   0.003   0.397
B A  1JPPB                448    0.164  18.084  -2.123   0.321   0.003   0.675
E R  1JPPB                449    0.512 117.294  -0.628   0.176   0.004   0.820
E D  1JPPB                450    0.363  52.323  -0.126   0.109   0.005   0.886
E V  1JPPB                451    0.639  98.245  -0.572   0.694   0.003   0.303
E H  1JPPB                452    0.688 125.056  -0.186   0.782   0.003   0.216
B N  1JPPB                453    0.158  23.117  -0.882   0.858   0.002   0.139
B R  1JPPB                454    0.197  45.205   0.156   0.923   0.002   0.076
E I  1JPPB                455    0.498  92.149   1.014   0.970   0.001   0.030
E V  1JPPB                456    0.269  41.284   1.284   0.970   0.001   0.030
B I  1JPPB                457    0.044   8.140  -0.353   0.970   0.001   0.030
E R  1JPPB                458    0.330  75.684   0.353   0.938   0.007   0.055
E G  1JPPB                459    0.661  52.005   0.254   0.879   0.010   0.111
E L  1JPPB                460    0.387  70.860  -0.080   0.600   0.003   0.397
E N  1JPPB                461    0.526  76.977  -1.125   0.321   0.003   0.675
E T  1JPPB                462    0.309  42.844  -0.356   0.406   0.004   0.590
B I  1JPPB                463    0.187  34.521   0.194   0.858   0.002   0.139
E P  1JPPB                464    0.575  81.578   0.372   0.923   0.002   0.076
B L  1JPPB                465    0.211  38.634   0.937   0.975   0.003   0.022
B F  1JPPB                466    0.025   5.058   0.259   0.970   0.001   0.030
B V  1JPPB                467    0.215  33.061   0.915   0.975   0.003   0.022
E Q  1JPPB                468    0.487  87.050   1.264   0.938   0.007   0.055
B L  1JPPB                469    0.080  14.556  -0.158   0.858   0.002   0.139
B L  1JPPB                470    0.112  20.452  -1.438   0.502   0.002   0.495
E Y  1JPPB                471    0.651 139.183   0.145   0.176   0.004   0.820
E S  1JPPB                472    0.527  61.706  -0.143   0.016   0.005   0.979
E P  1JPPB                473    0.708 100.465  -1.873   0.003   0.003   0.994
E I  1JPPB                474    0.475  87.819  -0.004   0.003   0.003   0.994
E E  1JPPB                475    0.613 107.056   0.451   0.923   0.002   0.076
E N  1JPPB                476    0.538  78.763   1.561   0.970   0.001   0.030
B I  1JPPB                477    0.045   8.306   0.446   0.970   0.001   0.030
B Q  1JPPB                478    0.098  17.431   1.226   0.970   0.001   0.030
E R  1JPPB                479    0.409  93.730   1.108   0.970   0.001   0.030
B V  1JPPB                480    0.222  34.198   1.611   0.970   0.001   0.030
B A  1JPPB                481    0.016   1.730   1.292   0.970   0.001   0.030
B A  1JPPB                482    0.045   4.959   0.093   0.970   0.001   0.030
E G  1JPPB                483    0.269  21.202   1.079   0.988   0.000   0.012
B V  1JPPB                484    0.036   5.549   0.625   0.988   0.000   0.012
B L  1JPPB                485    0.020   3.662   1.224   0.988   0.000   0.012
B C  1JPPB                486    0.141  19.740   0.250   0.970   0.001   0.030
E E  1JPPB                487    0.319  55.782   1.069   0.938   0.007   0.055
B L  1JPPB                488    0.059  10.766   0.927   0.879   0.010   0.111
B A  1JPPB                489    0.056   6.138  -0.980   0.406   0.004   0.590
E Q  1JPPB                490    0.492  87.853   0.140   0.109   0.005   0.886
E D  1JPPB                491    0.401  57.842   0.539   0.016   0.005   0.979
E K  1JPPB                492    0.652 134.137  -0.009   0.600   0.003   0.397
E E  1JPPB                493    0.747 130.431   0.846   0.694   0.003   0.303
B A  1JPPB                494    0.114  12.585   0.040   0.858   0.002   0.139
B A  1JPPB                495    0.143  15.748   1.156   0.938   0.007   0.055
E E  1JPPB                496    0.538  94.076   1.614   0.970   0.001   0.030
B A  1JPPB                497    0.222  24.431   2.081   0.970   0.001   0.030
B I  1JPPB                498    0.028   5.235   0.406   0.975   0.003   0.022
E E  1JPPB                499    0.278  48.514   1.002   0.938   0.007   0.055
E A  1JPPB                500    0.693  76.325   0.889   0.879   0.010   0.111
E E  1JPPB                501    0.414  72.291   0.445   0.406   0.004   0.590
E G  1JPPB                502    0.540  42.498  -0.292   0.053   0.005   0.942
B A  1JPPB                503    0.071   7.824  -0.144   0.321   0.003   0.675
B T  1JPPB                504    0.110  15.257   1.110   0.923   0.002   0.076
E A  1JPPB                505    0.546  60.169   1.363   0.975   0.003   0.022
B P  1JPPB                506    0.215  30.551   1.566   0.970   0.001   0.030
B L  1JPPB                507    0.022   4.047   1.069   0.988   0.000   0.012
B T  1JPPB                508    0.192  26.630   1.406   0.970   0.001   0.030
E E  1JPPB                509    0.523  91.298   1.740   0.938   0.007   0.055
B L  1JPPB                510    0.069  12.597   0.351   0.858   0.002   0.139
B L  1JPPB                511    0.073  13.293  -0.089   0.502   0.002   0.495
E H  1JPPB                512    0.684 124.420   1.155   0.176   0.004   0.820
E S  1JPPB                513    0.379  44.419   0.069   0.016   0.005   0.979
E R  1JPPB                514    0.726 166.208  -0.587   0.003   0.003   0.994
E N  1JPPB                515    0.471  69.013   0.193   0.016   0.005   0.979
E E  1JPPB                516    0.624 108.943   0.774   0.970   0.001   0.030
E G  1JPPB                517    0.546  42.947   1.552   0.970   0.001   0.030
B V  1JPPB                518    0.030   4.626   0.142   0.975   0.003   0.022
B A  1JPPB                519    0.106  11.692   1.181   0.970   0.001   0.030
E T  1JPPB                520    0.454  63.011   1.586   0.970   0.001   0.030
B Y  1JPPB                521    0.193  41.201   1.787   0.970   0.001   0.030
B A  1JPPB                522    0.014   1.521   1.440   0.970   0.001   0.030
B A  1JPPB                523    0.078   8.640   0.399   0.970   0.001   0.030
E A  1JPPB                524    0.334  36.763   1.459   0.970   0.001   0.030
B V  1JPPB                525    0.028   4.365   0.729   0.988   0.000   0.012
B L  1JPPB                526    0.020   3.754   1.169   0.988   0.000   0.012
B F  1JPPB                527    0.207  41.585   0.632   0.970   0.001   0.030
E R  1JPPB                528    0.304  69.547   1.478   0.970   0.001   0.030
B M  1JPPB                529    0.036   7.104   0.493   0.923   0.002   0.076
B S  1JPPB                530    0.099  11.591  -0.853   0.782   0.003   0.216
E E  1JPPB                531    0.556  97.046   0.563   0.502   0.002   0.495
E D  1JPPB                532    0.514  74.111  -0.070   0.321   0.003   0.675
E K  1JPPB                533    0.487 100.196   0.267   0.176   0.004   0.820
E P  1JPPB                534    0.510  72.412  -0.432   0.246   0.004   0.750
E Q  1JPPB                535    0.636 113.590  -0.092   0.176   0.004   0.820
E D  1JPPB                536    0.585  84.284   0.168   0.109   0.005   0.886
E Y  1JPPB                537    0.523 111.786  -0.114   0.115   0.016   0.868
E K  1JPPB                538    0.709 145.780   0.385   0.003   0.003   0.994

Explain the output.  Go back.
```

---
